# Supplementary material for: Hyaluronic acid fuels pancreatic cancer cell growth
Source: eLife. 2021 Dec 24;10:e62645. doi: 10.7554/eLife.62645 (PMC8730721; doi:10.7554/eLife.62645)
Supplement: Source data 1. [file elife-62645-supp1.docx]

**Figure 1–Source Data 1.** Raw western blot images for Figure 1B and Figure 1E.

**
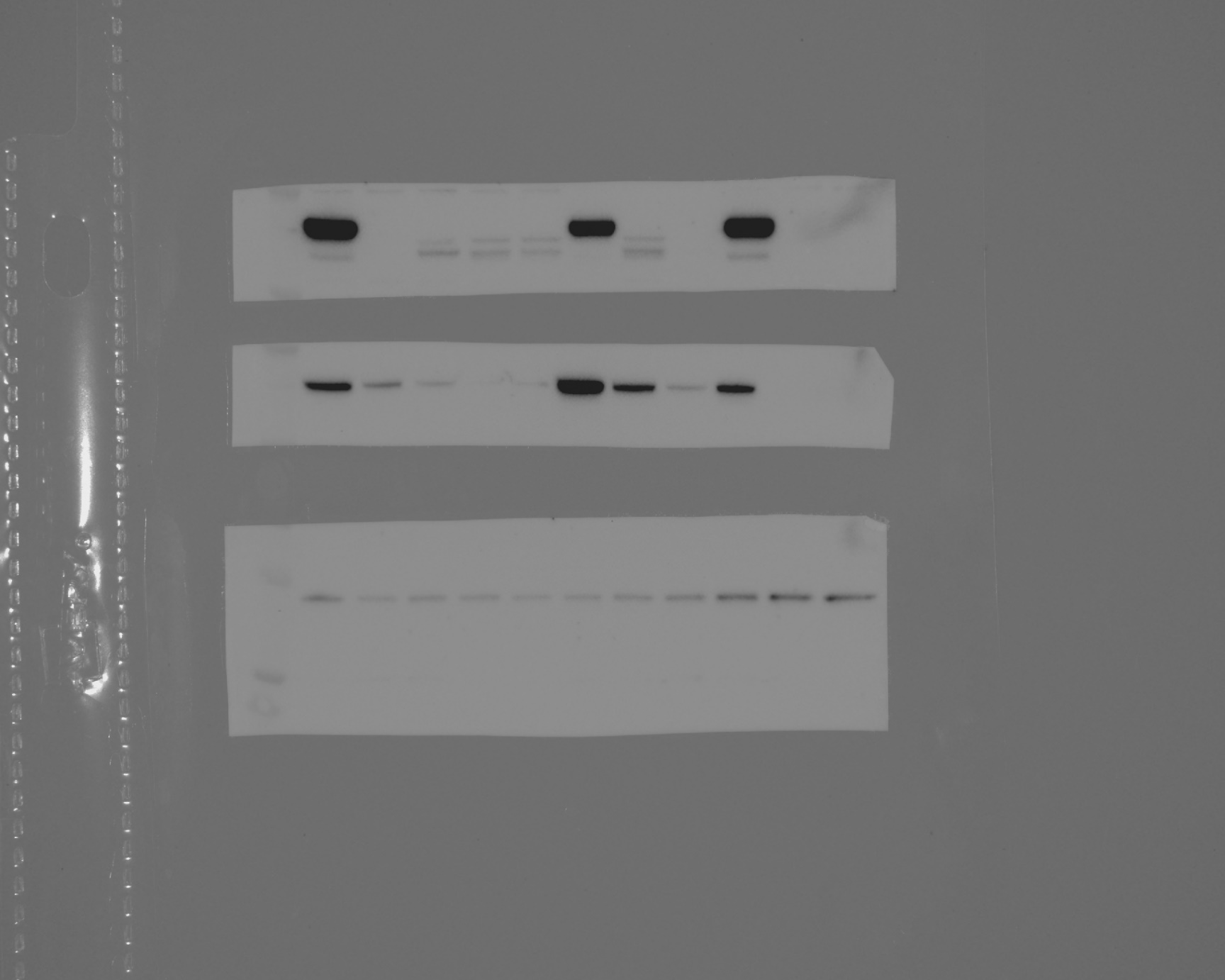
**


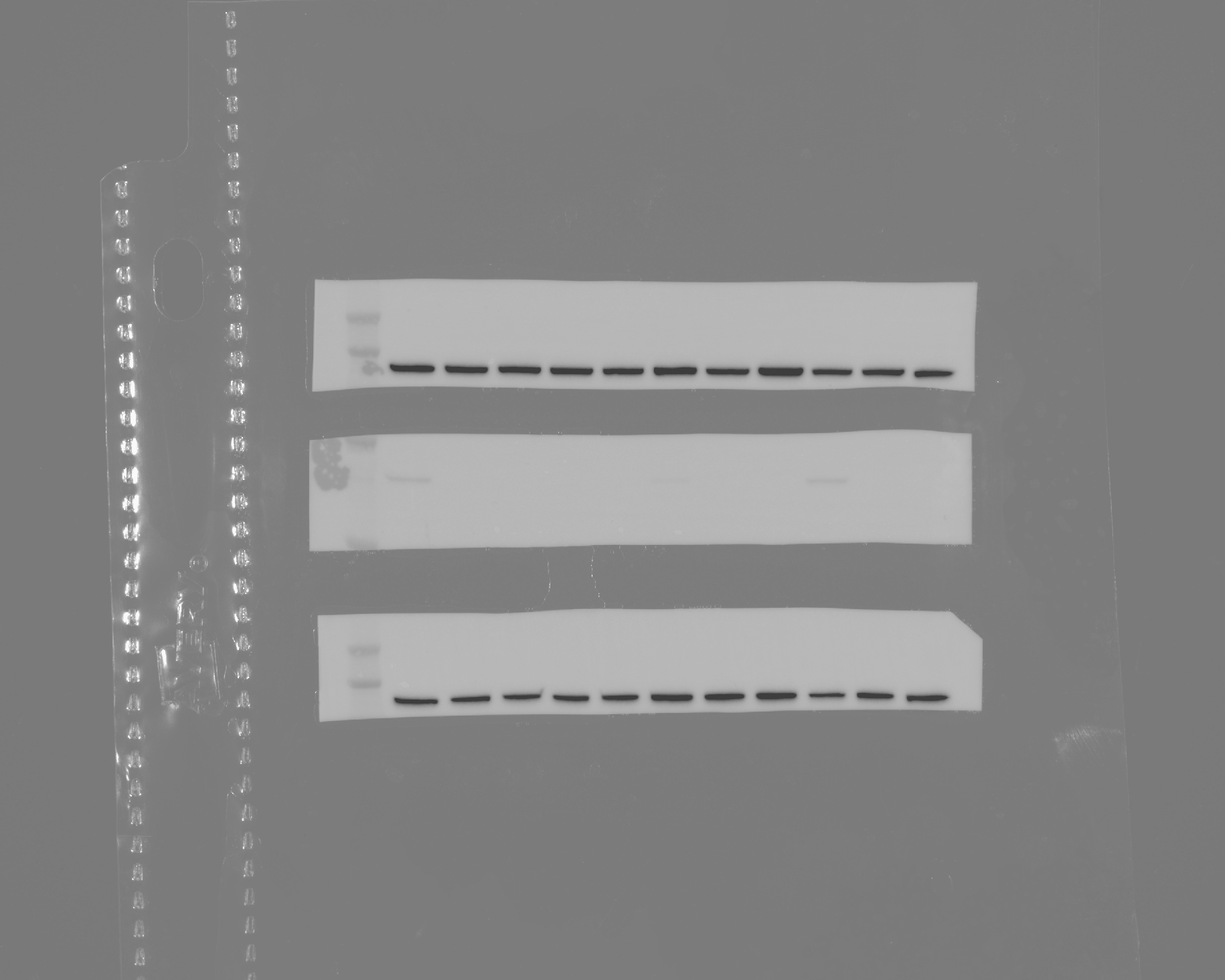
 TU8988T B9, MiaPaCa2 M12, HPAC H7: GFAT1

TU8988T B9, MiaPaCa2 M12, HPAC H7: VINCULIN (VNC)


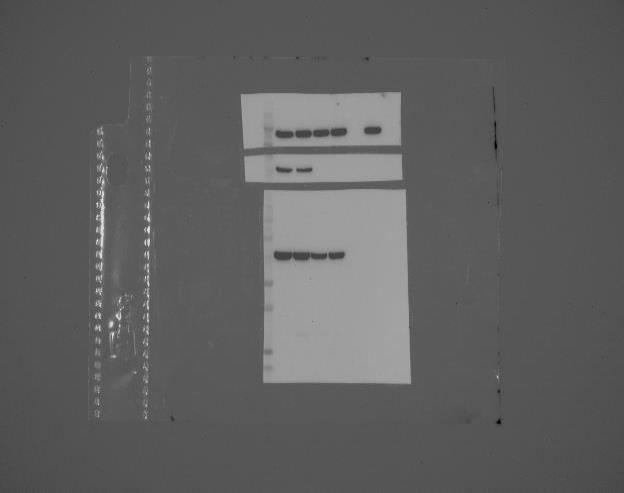


TU8988T tumors: GFAT1

**
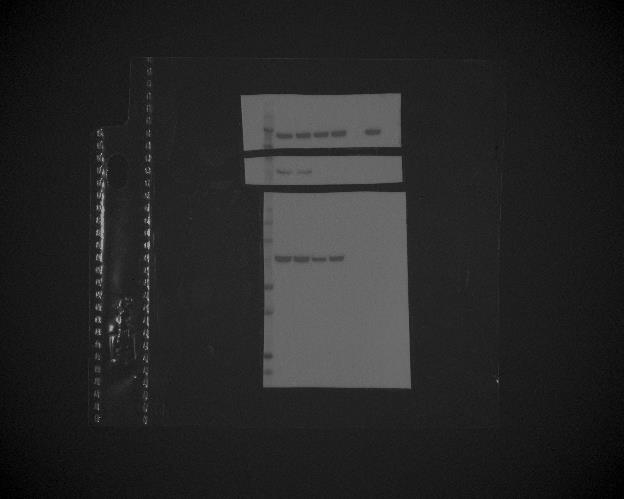
**

TU8988T tumors: VNC

**Figure 3–Source Data 1.** Raw western blot images for Figure 3C-E.

**O-GlcNAc**

**
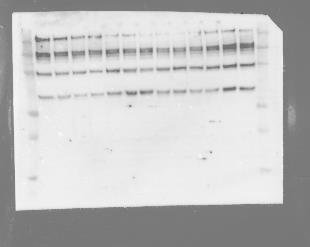
**

WT TU8988T


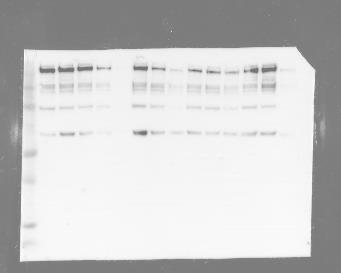

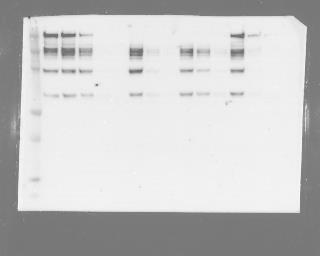


TU8988T B9 TU8988T D10

**VNC**


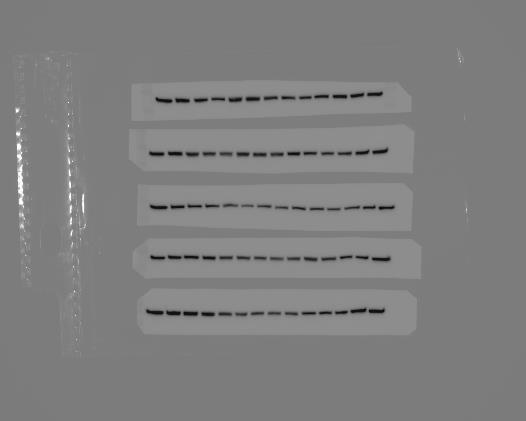


WT TU8988T

TU8988T B9

TU8988T D10

**Figure 5–Source Data 1.** Raw western blot images for Figure 5.


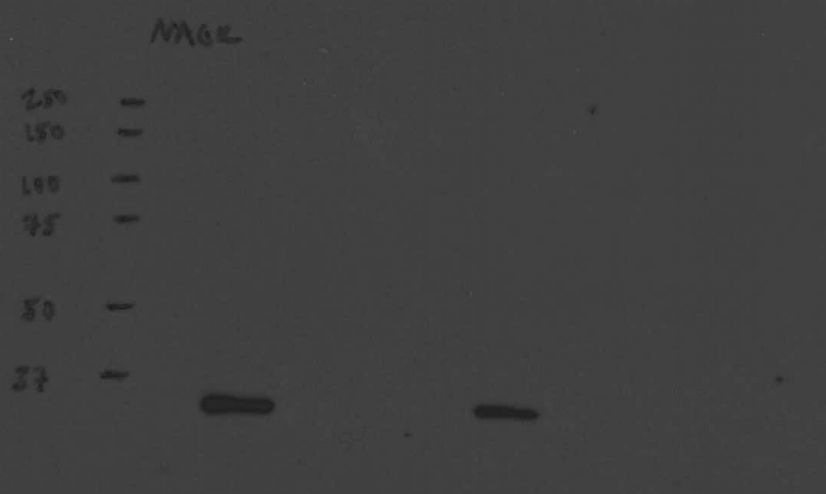


**NAGK**

Lane ID for “Figure 5A”

1. WT TU8988T
2. TU8988T NAGK KO sg1
3. TU8988T NAGK KO sg2
4. WT MiaPaCa2
5. MiaPaCa2 NAGK KO sg1
6. MiaPaCa2 NAGK KO sg2

1 2 3 4 5 6


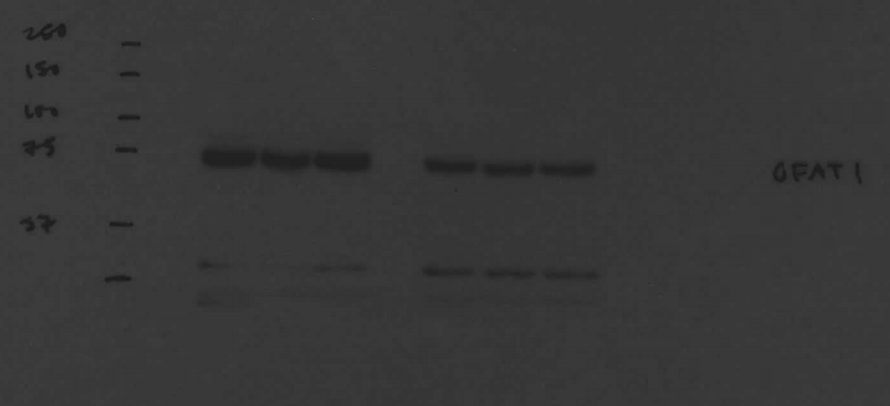


1 2 3 4 5 6

**GFAT1**


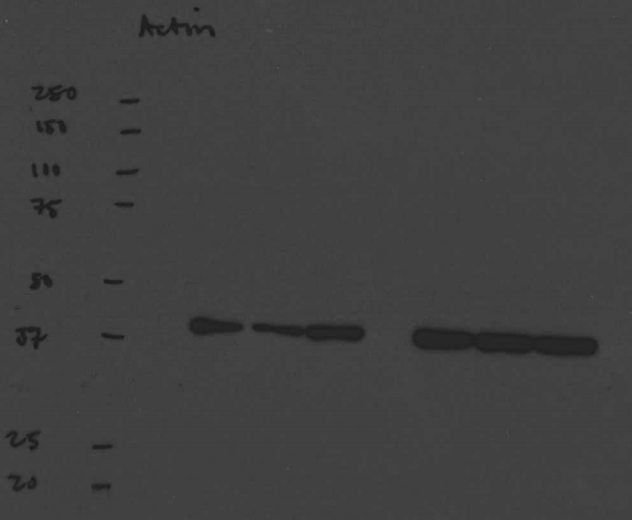


**ACTIN**

1 2 3 4 5 6

**
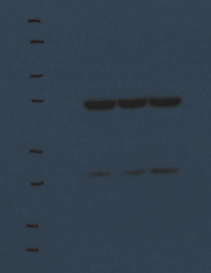

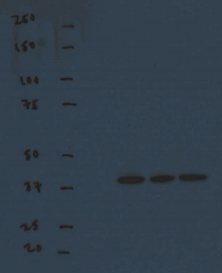

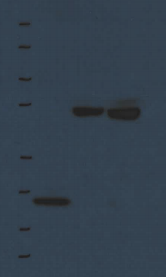
**

1 2 3

1 2 3

1 2 3

**NAGK GFAT1 ACTIN**

Lane ID for “Figure 5A continued”

1. WT HPAC
2. HPAC NAGK KO sg1
3. HPAC NAGK KO sg2

**
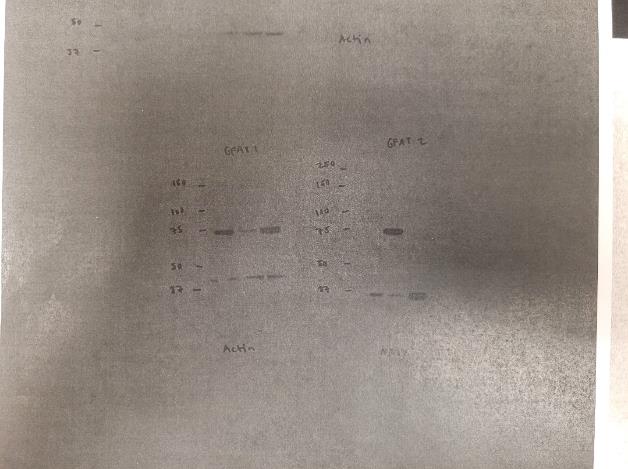

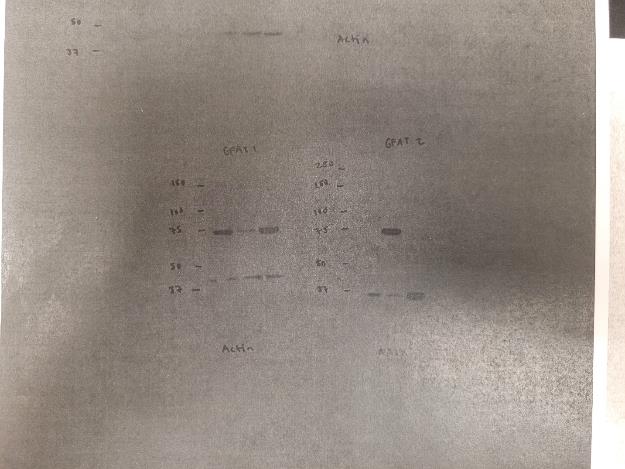
**

Lane 1: TU8988T

Lane 2: GFAT1

Lane 3: ACTIN

**GFAT1 NAGK**

**
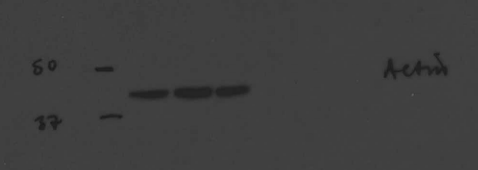
**

**ACTIN**

**
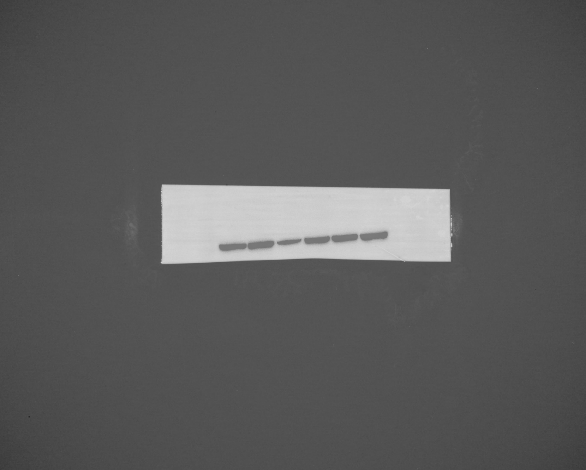
**

98

198

**Vinculin**

**
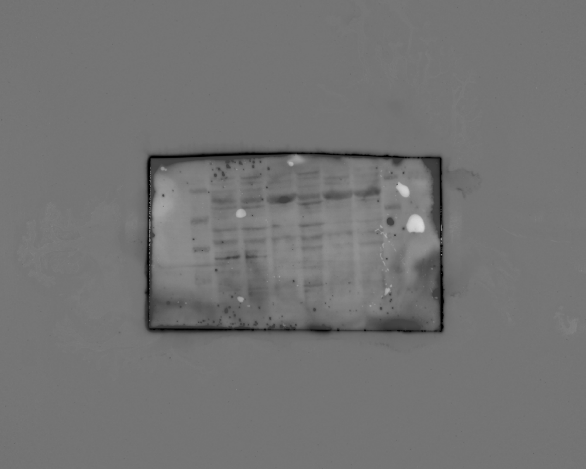
**

62

49

38

28

**NAGK**

**
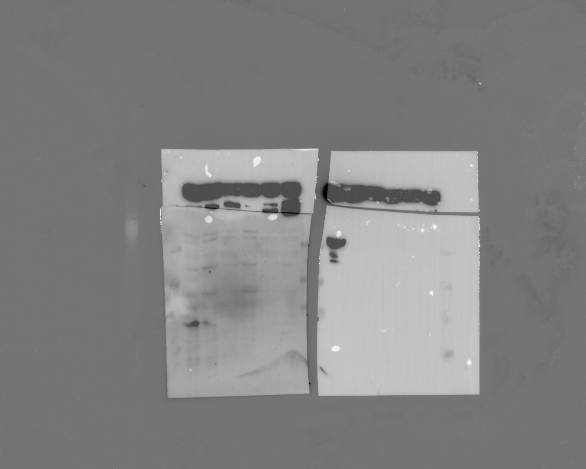
**

62

49

38

98

198

28

**Vinculin**

**GFAT1**


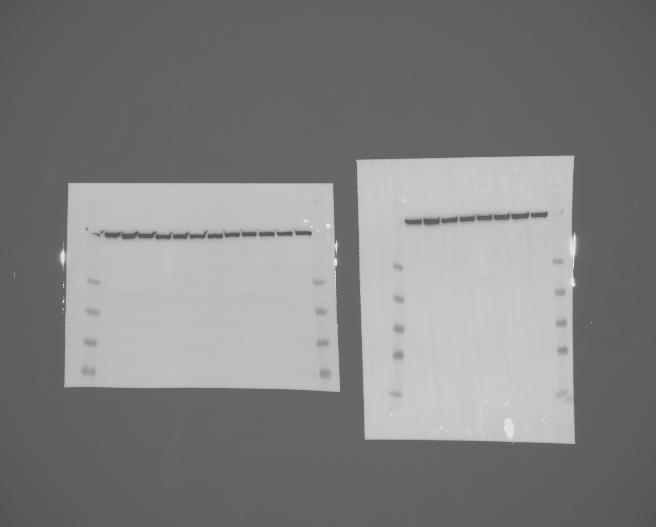


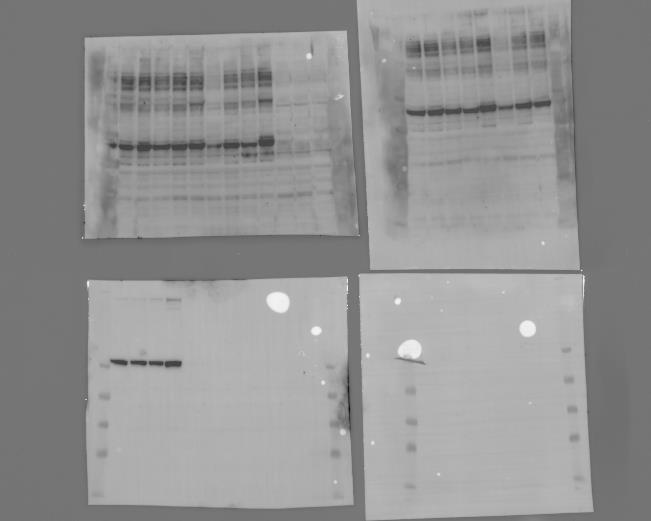

 **O-GlcNAc Vinculin**

**
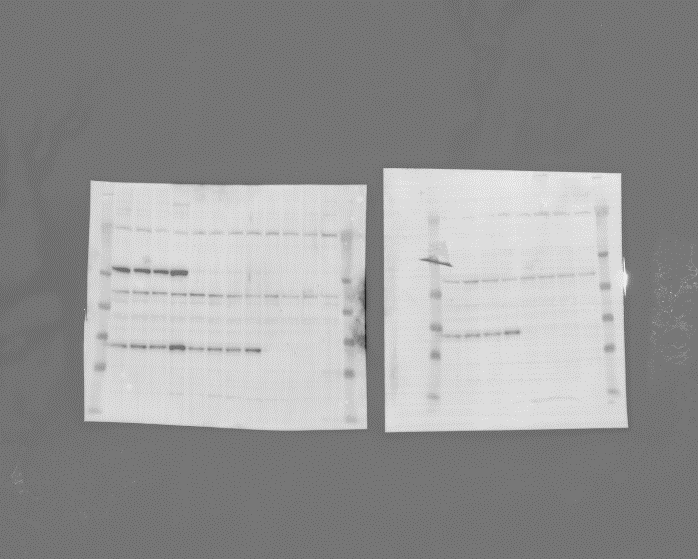
**

**NAGK**

**GFAT1 (probed first)**

**Figure 1–Source Data 2.** Raw western blot images for Figure 1–Figure Supplement 1D,E.

**
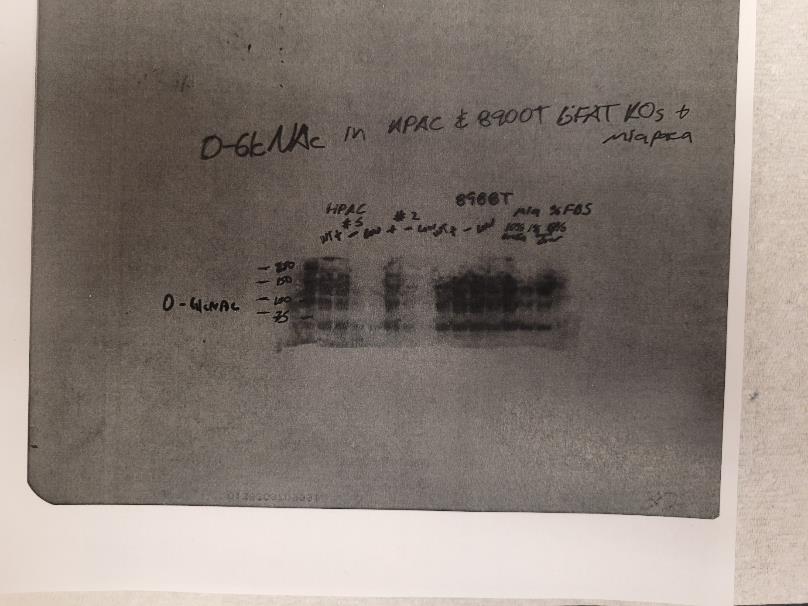
**

**
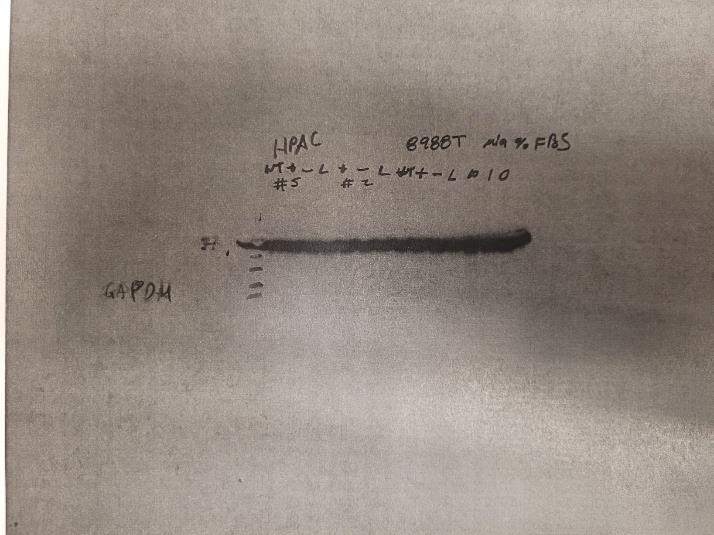
**

**
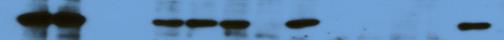

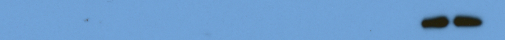
**

GFAT1 short

GFAT1 long

**
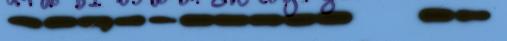
**

Actin

**Figure 5–Source Data 2.** Raw western blot images for Figure 5–Figure Supplement 1N.


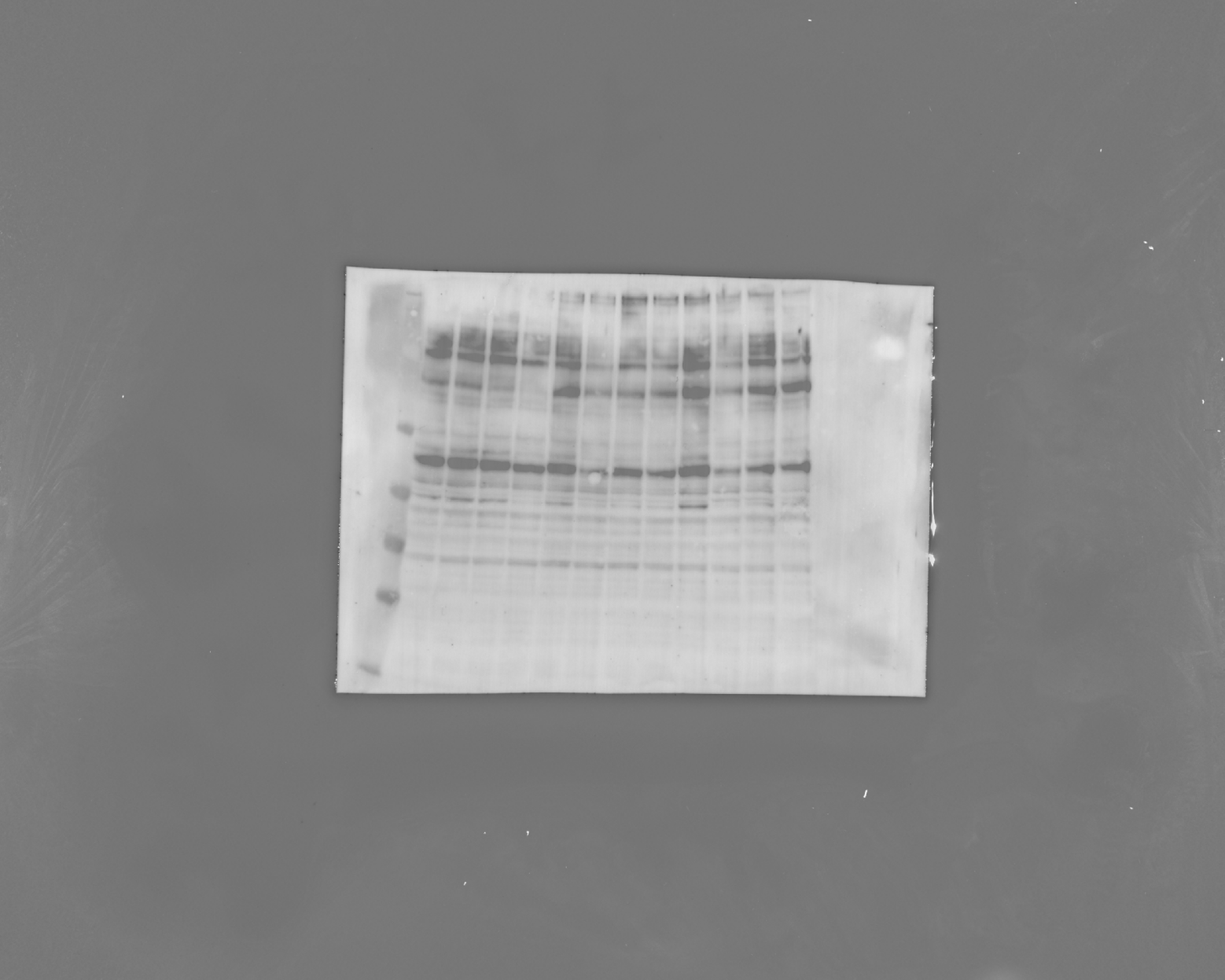


191

97

64

51

39

28


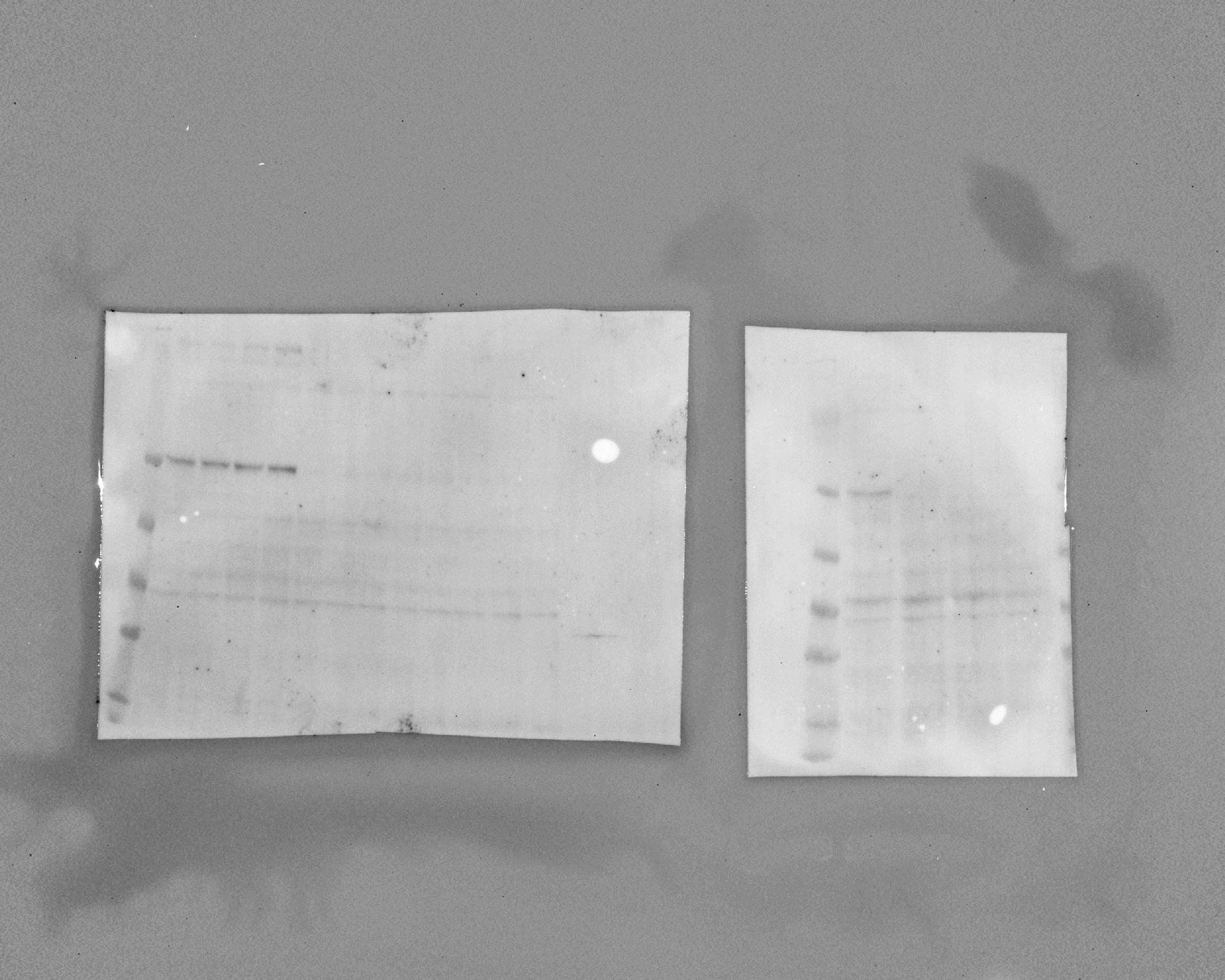


191

97

64

51

39

28

**O-GlcNAc GFAT1**


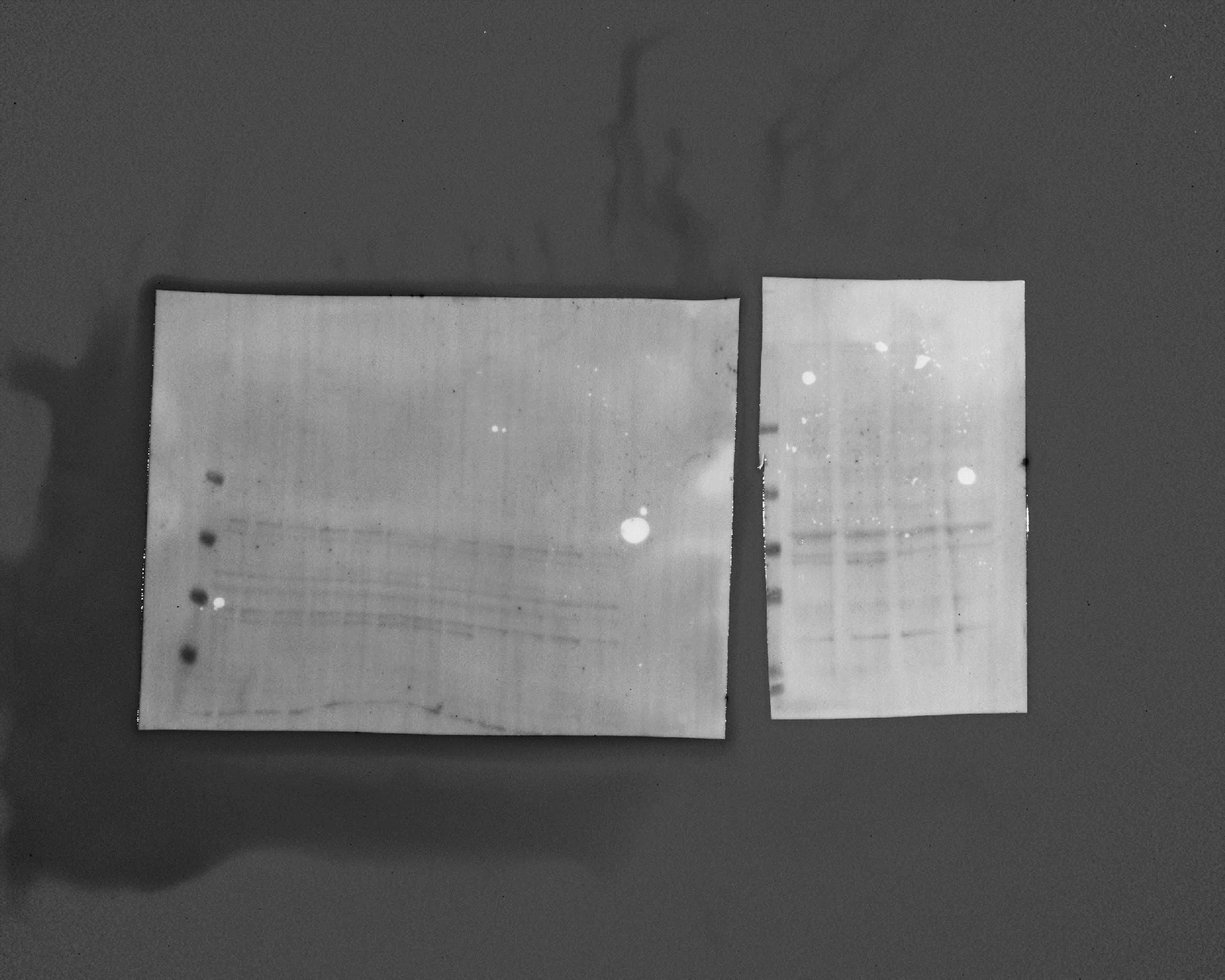


191

97

64

51

39

28


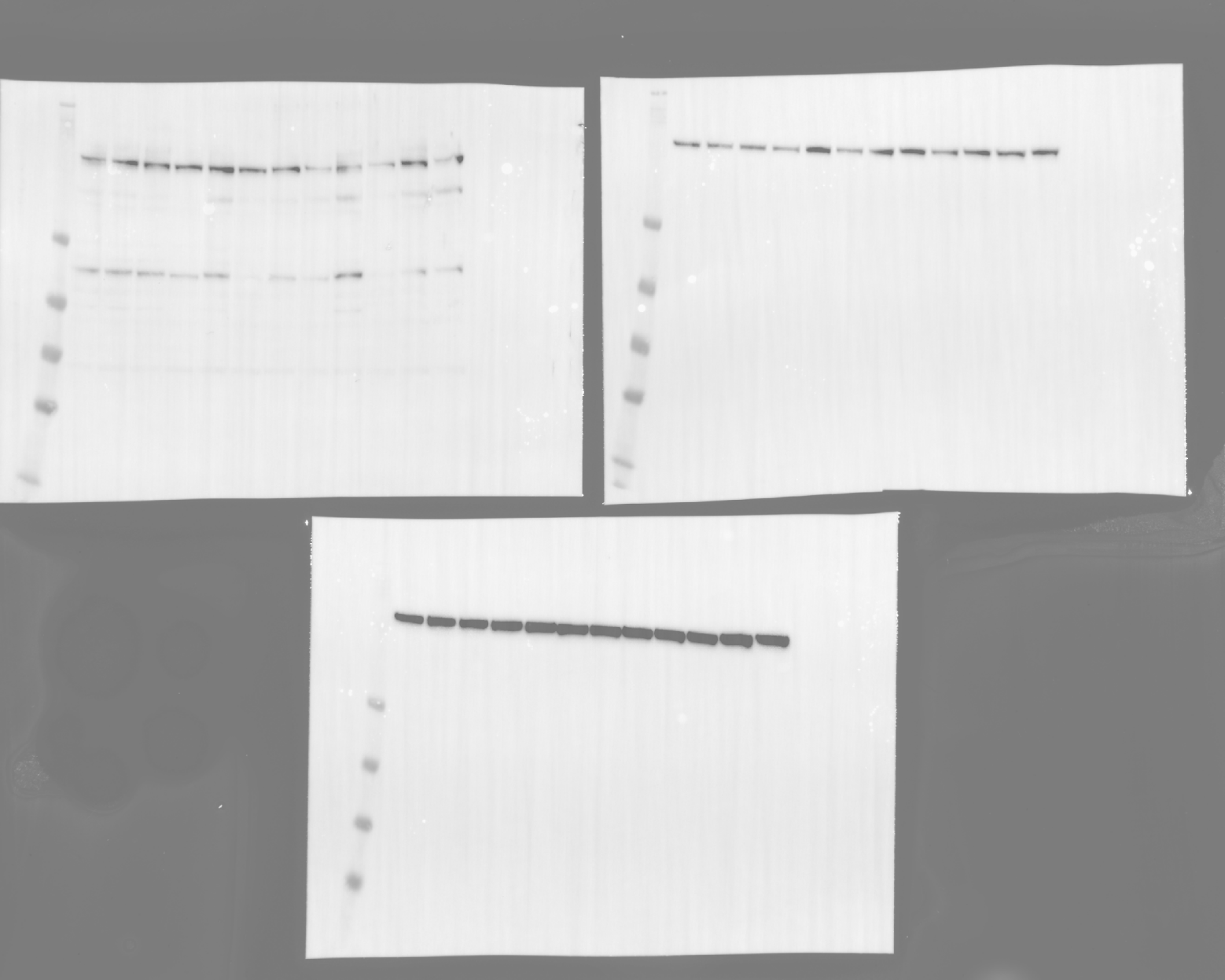


191

97

64

51

39

28

**NAGK VINCULIN**
